# The Exocyst Component Sec3 Controls Egg Chamber Development Through Notch During *Drosophila* Oogenesis

**DOI:** 10.3389/fphys.2019.00345

**Published:** 2019-03-29

**Authors:** Ping Wan, Sumei Zheng, Lai Chen, Dou Wang, Ting Liao, Xueming Yan, Xiaoji Wang

**Affiliations:** ^1^School of Life Science, Jiangxi Science and Technology Normal University, Nanchang, China; ^2^Experimental Animal Science and Technology Center, Jiangxi University of Traditional Chinese Medicine, Nanchang, China; ^3^Model Animal Research Center, Nanjing University, Nanjing, China

**Keywords:** Sec3, exocyst, Notch, *Drosophila*, ovary

## Abstract

The exocyst complex plays multiple roles via tethering secretory or recycling vesicles to the plasma membrane. Previous studies have demonstrated that the exocyst contains eight components, which possibly have some redundant but distinct functions. It is therefore interesting to investigate the biological function of each component. Here, we found that Sec3, one component of exocyst complex, is involved in *Drosophila* egg chamber development. Loss of *sec3* results in egg chamber fusion through the abolishment of cell differentiation. In addition, loss of *sec3* increases cell numbers but decreases cell size. These defects phenocopy Notch pathway inactivation. In line with this, loss of *sec3* indeed leads to Notch protein accumulation, suggesting that the loss of Sec3 inhibits the delivery of Notch onto the plasma membrane and accumulates inactive Notch in the cytoplasm. Loss of *sec3* also leads to the ectopic expression of two Notch pathway target genes, Cut and FasciclinIII, which should normally be downregulated by Notch. Altogether, our study revealed that Sec3 governs egg chamber development through the regulation of Notch, and provides fresh insights into the regulation of oogenesis.

## Introduction

Notch signaling is an evolutionarily conserved pathway that controls various processes, including embryogenesis and cell differentiation (Lai, 2004; Kopan and Ilagan, 2009). Vesicle-mediated protein traffic is essential for the transduction of the Notch pathway. The newly synthesized Notch receptor and DSL (Delta/Serrate/LAG-2) ligands are transported through the endoplasmic reticulum (ER) and Golgi apparatus to reach the plasma membrane. After association with ligands, the receptors re-enter the cell via endocytosis. These endocytic vesicles from the cell membrane then fuse with early endosome. The early endosome works as a sorting center, from which the Notch can be recycled back to the plasma membrane, or to the late endosome for protein degradation (Sorkin and von Zastrow, 2009).

Increasing studies on *Drosophila*, have demonstrated that the Notch pathway is involved in multiple processes, including ovary development. The *Drosophila* ovary consists of about 16–20 ovarioles, each of which contains a series of egg chambers processed through 14 stages. Each egg chamber consists of 16 germ cells enveloped by a monolayer of follicular epithelium. The neighboring egg chamber is linked by the stock, which is a string of five to eight follicle cells. A pair of special follicle cells called polar cells differentiate during early stages at each pole of each egg chamber. Loss of polar cells induces fusion of neighboring egg chambers and results in a compound egg chamber containing two or more germline clusters. Reduction of Notch activity suppresses polar cell formation and results in fused egg chambers (Grammont and Irvine, 2001; Torres et al., 2003; Vachias et al., 2010). Moreover, *Drosophila* oogenesis is a complex but coordinated process. For example, follicle cells switch from the mitotic cycle to endocycle during mid stages. Notch signaling induces this cell-cycle switch (Sun and Deng, 2005).

Exocyst is an eight protein complex and was originally identified from mutants involved in the secretory pathway in yeast (Novick et al., 1980). Subsequent analysis of the exocyst has shown that it functions in intracellular vesicle transport and mediates the tethering of secretory or recycling vesicles to the plasma membrane. Defects in exocyst proteins result in the accumulation of secretory vesicles in cells (Novick et al., 1980; Guo et al., 1999; Zhang et al., 2005). The exocyst components, Sec3, Sec5, Sec6, Sec8, Sec10, Sec15, Exo70, and Exo84, are conserved from yeast to mammals. In yeast, the Sec3 component functions as a spatial landmark for polarized secretion, as it localizes to the exocytic site independently of other components (Finger et al., 1998; Boyd et al., 2004). The yeast Sec3 directly binds to phosphatidylinositol 4,5-bisphosphate (PIP2) and the small GTPases Rho1 and Cdc42 through its N-terminal domain, and these bindings are critical for the localization and function of Sec3 at the exocytic site in the plasma membrane (Zhang et al., 2008; Yamashita et al., 2010). The yeast Sec3 was also found to interact with the t-SNARE protein Sso2, which promotes membrane fusion between the vesicles and target membrane (Yue et al., 2017). In plants, Sec3 has also been widely investigated. Sec3 is required for root hair elongation, embryogenesis, and pollen germination (Wen et al., 2005; Zhang et al., 2013; Bloch et al., 2016). In *Drosophila*, Sec3 is involved in the polarized transport of guidance receptors during border cell migration (Wan et al., 2013). However, the function of Sec3 in animals has not been well elucidated.

In this study, we show that the loss of *sec3* phenocopies loss-of-function Notch during *Drosophila* oogenesis. Further studies show that loss of *sec3* indeed results in a Notch protein transport defect and a Notch pathway inactivation, indicating that Sec3 regulates Notch signaling during oogenesis. Altogether, our findings shed light on exocyst-mediated regulation of oogenesis through Notch.

## Materials and Methods

### Fly Stocks and Husbandry

All fly stocks except for RNAi experiments were cultured at 25 ± 1°C on a standard corn-yeast-sucrose medium under constant humidity and a 12:12 h light to dark cycle. The *sec3*^GT^ and *sec3*^PBac^ lines were described previously (Wan et al., 2013). The line *hs-flp; ubi-GFP FRT80B* was obtained from the Bloomington *Drosophila* Stock Center (BDSC). Females of the latter line were crossed to males of *sec3*^GT^ and *sec3*^PBac^ lines, respectively. Progeny were heat shocked for 2 h per day at 37°C for 2 days before eclosion and 1 day after eclosion, then dissected 2–3 days after the last heat shock. Progeny with genotypes of *hs-flp; sec3^GT^ FRT80B/ubi-GFP FRT80B* or *hs-flp; sec3^PBac^ FRT80B/ubi-GFP FRT80B* were analyzed. The *sec3* mutant clones were marked by the absence of GFP. The *sec3* RNAi line was obtained from the Vienna *Drosophila* RNAi Center (#108085) and *c306-Gal4* from BDSC. The *c306-Gal4>sec3-RNAi* progeny were shifted to 29°C for 3–4 days before dissection.

### Immunohistochemistry and Microscopy

Ovary dissection was carried out in phosphate buffered saline (PBS) and then fixed in PBS with 7% formaldehyde for 10 min. After being washed in PBS, ovaries were blocked in 10% goat serum in PBT (PBS containing 0.3% Triton X-100) for 30 min and then stained overnight at 4°C. Most primary antibodies were obtained from the Developmental Studies Hybridoma Bank (DSHB) and their dilutions were as follows: mouse anti-Orb (orb 4H8, 1:30), mouse anti-NICD (C17.9C6, 1:10), mouse anti-NECD (C458.2H, 1:100), mouse anti-FASIII (7G10, 1:100), mouse anti-Cut (2B10, 1:100), mouse anti-Delta (C594.9B, 1:100), mouse anti-Kel (Kel 1B, 1:5), mouse anti-β-Gal (40-1a, 1:25), mouse anti-CycA (A12, 1:15), rabbit anti-phosho-Histone H3 (1:1000, Cell Signaling #3377), and rabbit anti-Stau (1:1000, gift from St. Johnston D). After being washed in PBT, ovaries were incubated with secondary antibodies (Jackson ImmunoResearch) for 2 h at room temperature. Nuclei were labeled by DAPI (E607303, 1:50, Sangon Biotech) in the last half hour. Confocal images were performed on a Leica TCS SP8 confocal microscope.

### Fertility Test

To check the fertility status of the *c306>sec3.RNAi* females, pair matings were set between individual *c306>sec3.RNAi* females and wild type males in a series of vials. All vials were raised in 29°C and changed to new vials every day, to retain a good oogenesis status. Egg laying/females/day of the fourth day were counted. The ratio of hatched vs. all laid eggs were then calculated after 24 h. Progeny of late stages were also observed to check the calculation. For the experimental control, wild type females were crossed with wild type males.

### Quantification of Cell Number and Cell Size

Regions of mutant clones were chosen, while regions of their sister clones were chosen. Cell numbers and area were measured in ImageJ software (NIH) for each region. The mean number of cells was calculated as (nucleus number)/(clone area). The mean size of the nuclei was calculated as (nucleus area)/(nucleus number). The mean ratio is an average of ratios of each pair bar. Statistical analysis for the data set was done using paired Student’s *t*-test, using Prism 5 (GraphPad software, San Diego, CA, United States). Data comparisons were considered statistically significant if *p* < 0.05.

## Results

### Loss of *sec3* Results in Egg Chamber Fusion

**Figure 1:**
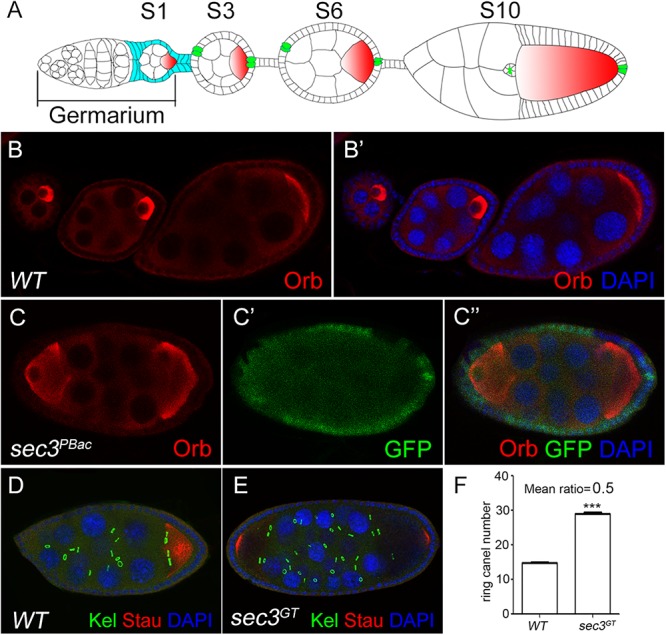
Loss of *sec3* in follicle cells results in egg chamber fusion. **(A)** A model for *Drosophila* oogenesis showing egg chambers in different stages. Oocytes are in red and polar cells are in green from stage 3. Follicle cells cover all the germline cysts at the early stages and mainly cover the oocyte by stage 10. **(B–B’)** A wild type ovariole with three egg chambers, each of which has an oocyte. **(C–C”)** A compound egg chamber with *sec3*^PBac^clones showing two oocytes. **(D)** A wild type egg chamber showing 15 ring canals. **(E)** A compound egg chamber with two oocytes showing 30 ring canals. **(F)** Quantification of ring canal numbers in compound egg chambers and control egg chambers (29.5 ± 0.2 vs. 14.8 ± 0.1, *n* = 12, *p* < 0.0001). Images for Kel staining are overlays of the Z-section, as ring canals cannot be shown in a plane. In all the images in this report, the *sec3* mutant clones are marked by an absence of GFP, and nuclei are stained by DAPI.

*sec3*^GT^ and *sec3*^PBac^ are two loss-of-function alleles of *Drosophila sec3* which were identified previously (Wan et al., 2013). *sec3* mutant follicle cell clones were generated with the Flp/FRT method and egg chambers were stained for the oocyte marker Orb. In the wild type, each egg chamber had only one Orb-positive oocyte and 15 nurse cells (Figure 1A–B’). In contrast, 12% (*sec3*^GT^) or 14% (*sec3*^PBac^) of the mutant mosaic egg chambers showed compound egg chambers, which had more than the normal complement of 15 nurse cells and an oocyte (*n* = 50) (Figure 1C–C” and Supplementary Figure S1). To confirm this, immunostaining of Kelch (Kel), a marker of ring canals, and Staufen (Stau), another marker of oocyte, was performed to count the number of germline cells in each egg chamber (Figure 1D,E). Ring canals are cytoplasmic bridges that intracellular materials can pass from nurse cells to oocyte. Every germline cell has one ring canal on average. Quantification results indicated that the germline cell number of these compound egg chambers was two copies of wild type (29.5 ± 0.2 vs. 14.8 ± 0.1, *n* = 12, *p* < 0.0001) (Figure 1F). Therefore, we hypothesized that loss of *sec3* led to egg chamber fusion. Some “partially” fused egg chambers undergoing the process of fusion could be observed, in which the intervening follicle cells seemed to be in the process of breaking apart (Supplementary Figures S2A–A”). The earliest “partially” fused egg chamber was observed at stage 3, when stock cells formed (Supplementary Figures S2B–B”). Most fused egg chambers were completely fused before stage 8, without an intervening wall of follicle cells between the two cysts.

### Knocking Down of *sec3* Suppresses Polar Cell Differentiation

**Figure 2:**
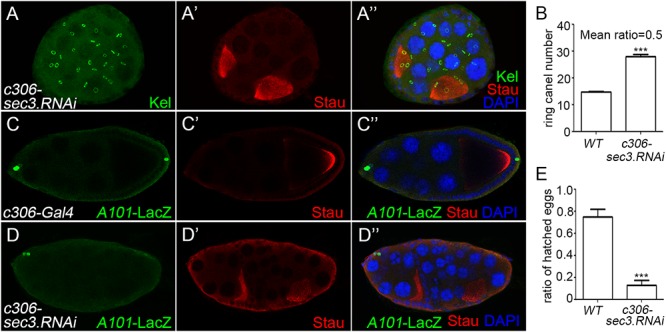
Knocking down of *sec3* induces a fused egg chamber and suppresses polar cell differentiation. **(A–A”)** In *c306>sec3-RNAi* background, a compound egg chamber with two oocytes shows 29 ring canals. Images for Kel staining are overlays of the Z-section, as ring canals cannot be shown in a plane. **(B)** Quantification of ring canal numbers in *sec3* knockdown compound egg chambers and control egg chambers (28.0 ± 0.7 vs. 14.8 ± 0.2, *n* = 8, *p* < 0.0001). **(C–C”)** In *c306-Gal4* background, *A101-LacZ* shows normal expression at the two poles of egg chamber. **(D–D”)** In *c306>sec3-RNAi* background, a partial fused egg chamber, which has no stock cells with only a single layer of follicle cells between the two cysts, contains only a pair of cells expressing *A101-LacZ* at only one pole of the egg chamber. Images for *A101*-LacZ staining are overlays of the Z-section, showing all the staining. **(E)** Fertility test of *c306>sec3-RNAi* females; see Methods for details. *c306>sec3-RNAi* females show a reduced ratio of hatched vs. all laid eggs (0.75 ± 0.07 vs. 0.13 ± 0.04, *n* = 14, *p* < 0.0001).

RNA interference was used to knock down *sec3* expression with the UAS/Gal4 method, using *c306-Gal4* which was expressed in most follicle cells. In *sec3* knocked down egg chambers, fused egg chambers were observed (Figure 2A–A”). Quantification results indicated that the germline cell number of the *sec3* knocked down compound egg chambers was two copies of the wild type (28.0 ± 0.7 vs. 14.8 ± 0.2, *n* = 8, *p* < 0.0001) (Figure 2B). The knock down experiment confirmed that reduced Sec3 activity induced egg chamber fusion. Signals from the polar cells are essential for stalk formation, and egg chamber fusion can be induced by suppressing polar cell differentiation (Baksa et al., 2002; McGregor et al., 2002). Polar cells in *sec3* knockdown egg chambers were examined, using an enhancer trap line *A101* (*neutralized-lacZ*), in which polar cells were marked with lacZ (Lopez-Schier and St Johnston, 2001; Figure 2C–C”). In *sec3* knocked down egg chambers, expression of *A101-lacZ* was reduced in fused egg chambers (Figure 2D–D” and Supplementary Figure S3). Therefore, reduced *sec3* activity resulted in the attenuation of polar cells, which contributed to the fusion of adjacent egg chambers.

A fertility test was carried out to test whether the dysfunction of the ovary was caused by the encapsulated multiple egg chambers. This was demonstrated by setting up pair matings between individual *c306>sec3.RNAi* females and wild type males. After being raised in 29°C for 4 days, egg laying/females/day and the ratio of hatched vs. all laid eggs were quantified. Most of the laid eggs from these pair matings remained unhatched and in some of the vials one to two eggs had hatched. Therefore, reduced *sec3* activity resulted in reduced fertility (Figure 2E).

### Loss of *sec3* Shows Dysregulated Cell Cycle

**Figure 3:**
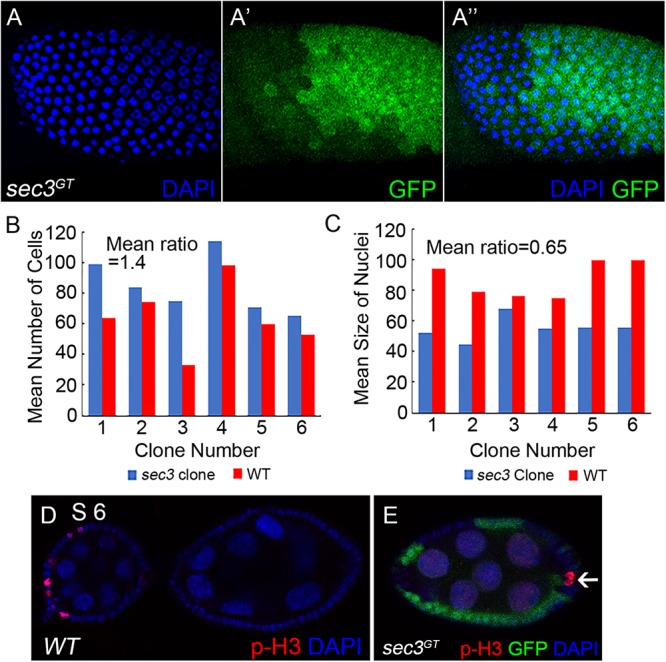
Loss of *sec3* disrupts the mitotic cycle/endocycle switch. **(A–A”)** In a stage 10 egg chamber, *sec3* mutant follicle clones show more cell numbers and smaller nuclei. Images are overlays of Z-section, as nuclei cannot be shown in a plane. **(B)** Number of nuclei in *sec3* mutant clones (blue bars) compared with that in their associated sister clones (red bars) in stage 10 egg chambers. The *x*-axis represents the clone number, and the *y*-axis represents the number of cells per clone or corresponding sister clone. Mean ratio of mutant/wt = 1.4, *n* = 6, *p* = 10^-2^. See Methods for details. **(C)** Quantification of mean nucleus size in *sec3* mutant clones and their sister clones. Mean ratio of mutant/wt = 0.65, *n* = 6, *p* = 3 × 10^-2^. See Methods for details. **(D)** In wild type egg chambers, p-H3 positive follicle cells are detected only up to stage 6. Note that while mitosis of follicle cells is not synchronized, p-H3 are detected only in some cells. **(E)** In *sec3* mutant follicle cells, p-H3 staining can be detected after stage 6.

During *Drosophila* oogenesis, follicle cells cease mitosis and duplicate chromosomes without cell division to generate polyploidy (endocycle) after stage 6 (Sun and Deng, 2005). The mitotic cycle/endocycle switch was then detected in *sec3* mosaic egg chambers. Follicle cells in stage 10 egg chambers were examined. Follicle cells in *sec3* mutant clones had obvious smaller nuclei, and were more densely distributed, compared with cells in the neighboring sister clone (Figure 3A–A”). The quantification results of mean cell number supported that mutant clones had more cell numbers than their sister clones (mutant/wt = 1.4, *n* = 6, *p* = 10^-2^) (Figure 3B). It suggested that *sec3* mutant cells failed to cease mitosis and processed extra rounds of cell division. The quantification results of mean nuclei size confirmed that mutant clones had smaller nuclei than their sister clones (mutant/wt = 0.65, *n* = 6, *p* = 3 × 10^-2^) (Figure 3C), which suggested that *sec3* mutant cells did not reach the endocycle.

Immunostaining results of the mitotic marker phospho-histone H3 (p-H3) also demonstrated that *sec3* mutant follicle cells continued to divide after stage 6. In wild type egg chambers, p-H3 positive follicle cells were detected only up to stage 6 during their mitotic cycle (Figure 3D). Note that while mitosis of follicle cells was not synchronized, p-H3 was detected only in some cells. In *sec3* mutant follicle cells p-H3 staining was observed after stage 6 (Figure 3E and Supplementary Figure S4).

### Notch Protein Traffic Is Destroyed in *sec3* Mutant Follicle Cells

**Figure 4:**
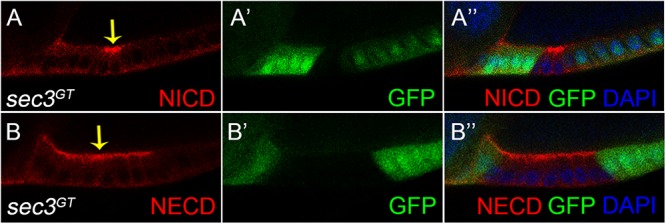
Accumulated Notch protein in *sec3* mutant follicle cells. **(A–A”)** NICD obviously accumulates in *sec3* mutant follicle cells in a stage 10 egg chamber (indicated by arrow). **(B–B”)** NECD obviously accumulates in *sec3* mutant follicle cells in a stage 10 egg chamber (indicated by arrow).

Both the fused eggs chamber and dysregulated cell proliferation phenocopied that of the Notch mutant clone (Deng et al., 2001; Grammont and Irvine, 2001; Vachias et al., 2010). There was therefore a need to examine whether Sec3 was involved in the traffic of Notch protein, as expected for a functional component of the exocyst complex. Without the exocyst, vesicles could not tether to the cell membrane which resulted in accumulation of vesicles and their cargo protein in the cytoplasm. The Notch receptor is a heterodimer composed of a Notch Intracellular Domain (NICD) and a Notch Extracellular Domain (NECD). In mosaic egg chambers, accumulation of NICD and NECD were specifically shown in the *sec3* mutant follicle cells, compared with the neighboring wild type follicle cells (Figure 4). Therefore, loss of Sec3 function might inhibit the delivery of Notch onto the plasma membrane, causing an accumulation of Notch protein in the cytoplasm, suggesting that Sec3 is essential for the delivery of Notch onto he plasma membrane.

### Notch Signaling Target Genes Are Falsely Expressed in *sec3* Mutant Follicle Cells

**Figure 5:**
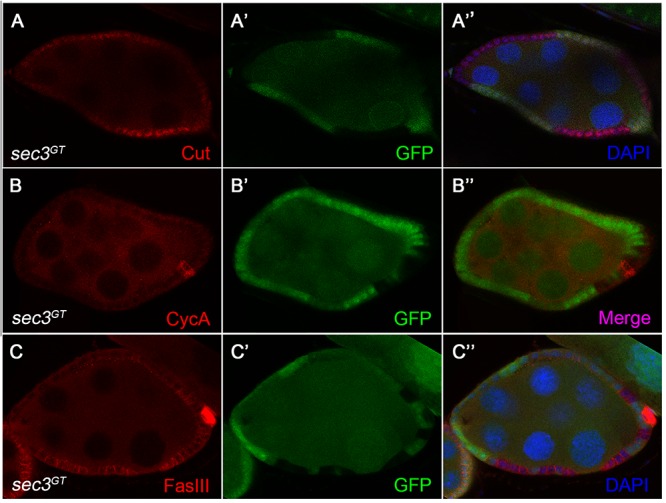
Altered expression of Notch signaling target genes in *sec3* mutant follicle cells. **(A–A”)** *sec3* mutant clones show continued expression of Cut, in contrast to the wild type follicle cells. **(B–B”)** *sec3* mutant clones show continued expression of CycA, in contrast to the wild type follicle cells. Note that while CycA was detected in 50% of follicle cells in the wild type egg chambers during stages 4–6, CycA was not uniformly expressed in mutant clones. **(C–C”)** *sec3* mutant clones show continued expression of FasIII, in contrast to the wild type follicle cells.

Cut, a DNA binding protein containing homeodomain, links Notch signaling and cell-cycle regulators. Cut is expressed in follicle cells during the early stages and is downregulated by the Notch signaling during the mid stages, which results in the cessation of mitosis and entry into the endocycle (Sun and Deng, 2005). We found that, in contrast to the wild type follicle cells, *sec3* mutant follicle cells showed continued expression of Cut beyond stage 6 (Figure 5A–A”). Consistent with Cut expression, CycA, the mitotic cyclin, continued to be expressed in mutant clones after stage 6, whereas wild type follicle cells demonstrated no CycA expression during these stages. Note that while CycA was detected in 50% of follicle cells in wild type egg chambers during stages 4–6, CycA was not uniformly expressed in mutant clones (Figure 5B–B”; Shcherbata et al., 2004).

Notch signaling also regulates the expressions of immature cell-fate markers in follicle cells. For example, in wild type egg chambers, FasciclinIII (FasIII) is expressed in all follicle cells during the early stages and then only expressed in two pairs of polar follicle cells at each end of the egg chamber by stage 4. This downregulation of FasIII marks the differentiation of the follicle cell. Reduced Notch activity catches follicle cells in an undifferentiated state and induces ectopic expression of FasIII at the late stages (Lopez-Schier and St Johnston, 2001). We observed strong expression of FasIII in cells mutant for *sec3* even after stage 6, suggesting that they were defective in Notch signaling (Figure 5C–C”). FasIII is also used as a marker of polar cells. However, mutant follicle cells failed to down regulate FasIII and should not be polar cells. Eyes absent (Eya) is normally detected in main-body follicle cells and absent from both polar cells and stalk cells during stage 1 to stage 8 (Bai and Montell, 2002). Immunostaining of Eya was performed and lack of Eya was not detected in mutant main-body follicle clones during stage 1 to stage 8 (Supplementary Figure S5).

## Discussion

This report demonstrates a role of *sec3* in regulating egg chamber development in *Drosophila* through Notch. This conclusion is based on results that show that: (1) a loss of *sec3* results in abnormal egg chamber development, which phenocopies Notch pathway inactivation; (2) notch accumulates in *sec3* clones; (3) target genes of Notch signaling are mis-expressed in *sec3* clones. The exocyst has been reported to have functions in the traffic of several membrane proteins, including the adhesion molecule, receptor, and the glucose transporter. However, in this study, the defect in the traffic of Notch protein does not seem to be a general one, as the exocyst is not required for all secretory events. For instance, the exocyst promotes recycling and tethering of E-Cadherin (E-Cad) containing vesicles in the *Drosophila* ovary or wing, but does not affect the distribution pattern and amount of another two polarity molecules, Baz/Par-3, and Dlg1 (Langevin et al., 2005; Wan et al., 2013). The exocyst is not required for synaptic vesicle release at mature synapses (Murthy et al., 2003). Fibrocystin, Polycystin-2, and Smoothened are transmembrane receptors localized in the ciliary membrane. The exocyst complex regulates the delivery of Fibrocystin and Polycystin-2 to the cilium, but not Smoothened (Monis et al., 2017). Moreover, Delta, the ligand of Notch, is not accumulated in *sec3* mutant cells (Supplementary Figure S6).

The positioning of the oocyte requires the upregulation of the E-cad in both the oocyte and the posterior follicle cells, which causes them to adhere to each other (Godt and Tepass, 1998; Gonzalez-Reyes and St Johnston, 1998). Although, it has been reported that E-cad accumulates in *sec3* mutant follicle cells (Wan et al., 2013), E-cad is not required for oocyte determination (Godt and Tepass, 1998; Gonzalez-Reyes and St Johnston, 1998), the change of protein levels of E-cad in follicle cells can change the position of the oocyte, but could not generate more than one oocyte in an egg chamber. Therefore, the compound egg chamber phenotype is not a consequence of abnormal E-cad intracellular traffic.

Recent studies have revealed a sophisticated regulation of the Notch receptor by vesicle trafficking. Endocytosis is important in Notch signaling as genetic interactions have been found between Notch and *shibire*, the *Drosophila* homolog of dynamin, a key regulator of endocytosis. *shibire* mutant cells may fail to internalize Notch and show Notch accumulation on the cell surface (Lu and Bilder, 2005). The Notch accumulation phenotype is also found in mutants in *avl* (*avalanche*) and *rab5*, two genes required for maturation of early endosomes (Yan et al., 2009). Active receptors can be internalized to the lysosome and degraded, which is a common mechanism of desensitization. Both NICD and NECD are detected in late endosomal compartments in *Drosophila* (Kooh et al., 1993). Rab11, a GTPase on the recycling endosomes and Sec15, another component of the exocyst complex, have functions in Delta recycling in the development of the *Drosophila* sensory organ precursor (SOP) cell lineage (Emery et al., 2005; Jafar-Nejad et al., 2005). In this study, we suppose that the accumulated Notch protein may be in recycling vesicles or in vesicles that transport from the post-Golgi to the plasma membrane. A shortcoming of this study is that we were not able to identify what kind of vesicle the Notch protein accumulated in.

Multiple intercellular signaling pathways involved in the steps of egg chamber development, and a group of mutations have shown an egg chamber fusion phenotype. In particular, reduction in *Notch* or *Delta* activity suppresses polar cell formation and results in fused egg chambers (Xu et al., 1992; Bender et al., 1993; Grammont and Irvine, 2001; Lopez-Schier and St Johnston, 2001). The polar cells signal through the JAK/STAT pathway to induce the formation of the stalk which separates adjacent cysts. Reduced JAK/STAT pathway activity results in fused egg chambers (Baksa et al., 2002; McGregor et al., 2002). The *Drosophila* gene *brainiac* (*brn*) and *egghead* (*egh*) show an identical egg chamber fusion phenotype. But they are required in the germline and not essential for differentiation of polar and stalk cells (Goode et al., 1996). The activity of the *hedgehog* (*hh*) gene stimulates the proliferation of pre-follicle somatic cells in the germarium. Reduced activity of *hh* produces compound egg chambers which result from a failure in the package of the germline cysts by somatic cells (Forbes et al., 1996). Egg chamber fusion in the *sec3* mosaic is not due to abnormal *hh* signaling, since *sec3* mutant clones can encapsulate germline cysts normally at stage 1 (Supplementary Figure S7). Data in this report support that the egg chamber fusion phenotype of *sec3* is due to the failure in polar cell formation for abnormal Notch activity.

In summary, this report has identified the developmental function of Sec3 and its link with Notch during *Drosophila* oogenesis. To our knowledge, this is the first report to identify a link between Sec3 and Notch. Components of the exocyst are conserved from yeast to a human, so it is conceivable that Sec3, as well as other components of the exocyst, are involved in regulating Notch in tissues of other species.

## Author Contributions

XW and PW designed the experiments. PW, SZ, LC, DW, and TL performed the data collection and analysis. PW and XY provided the fund. PW and XW wrote the manuscript.

## Conflict of Interest Statement

The authors declare that the research was conducted in the absence of any commercial or financial relationships that could be construed as a potential conflict of interest.
