# Supplementary material for: The Exocyst Component Sec3 Controls Egg Chamber Development Through Notch During Drosophila Oogenesis
Source: Front Physiol. 2019 Mar 29;10:345. doi: 10.3389/fphys.2019.00345 (PMC6450198; doi:10.3389/fphys.2019.00345)
Supplement: Supplementary file 1 [file Data_Sheet_1.PDF]

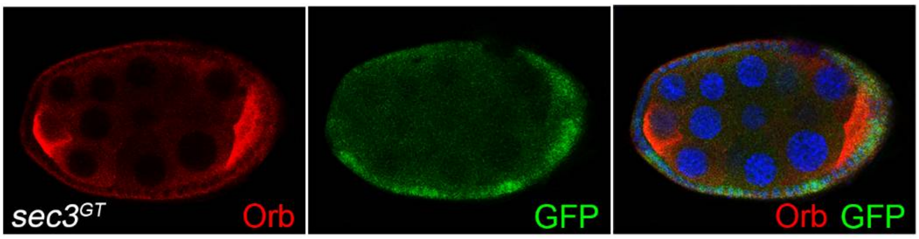

FIGURE S1. An example of compound egg chambers with *sec3*^{GT} clone. In all the pictures in this supplementary file, the *sec3* mutant clones are marked by absence of GFP, and nuclei are stained by DAPI.

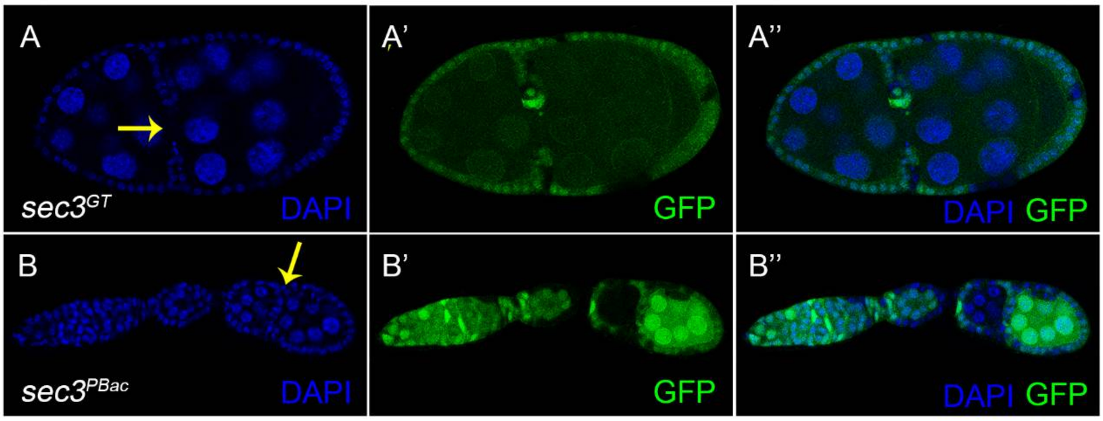

FIGURE S2. Examples of "partial" fused egg chambers. (A-A") A "partial" fused egg chamber was undergoing fusion. Arrow marks the gap formed in the double layer of follicle cells between the two cysts. (B-B") A "partial" fused egg chamber was formed at stage3 when stock cell formed. Arrow marks the intervening wall of follicle cells between the two cysts.

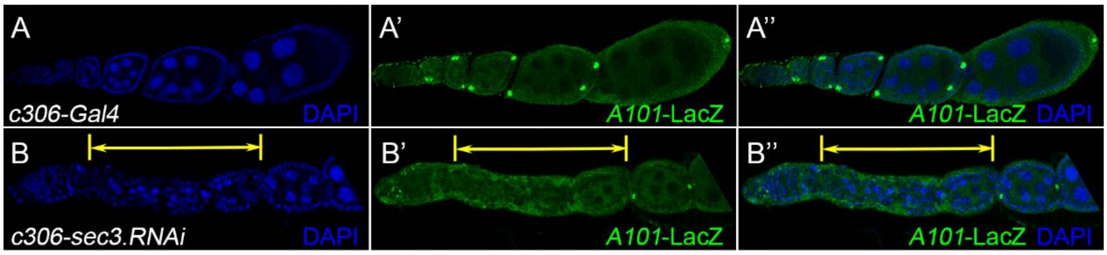

FIGURE S3. Knocking down of *sec3* suppresses polar cell differentiation. (A-A") In *c306-Gal4* background, *A101-LacZ* shows normal expression at the two poles of egg chambers. (B-B") In *c306>sec3-RNAi* background, an ovariole shows no *A101-LacZ* in fused egg chamber (figured out).

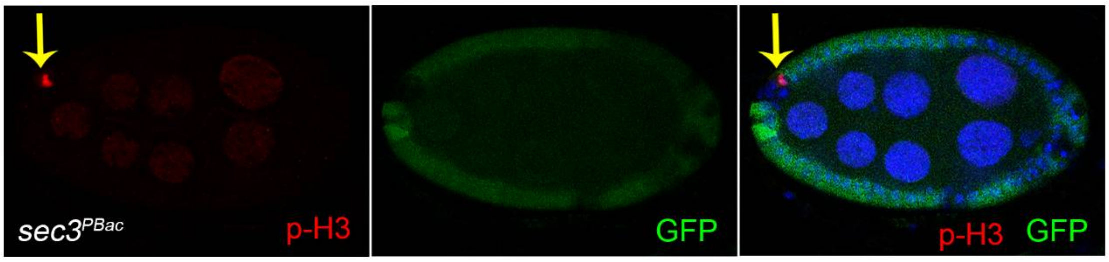

FIGURE S4. Staining of phospho-histone H3 (p-H3) was observed after stage 6. Arrow marks the staining of p-H3.

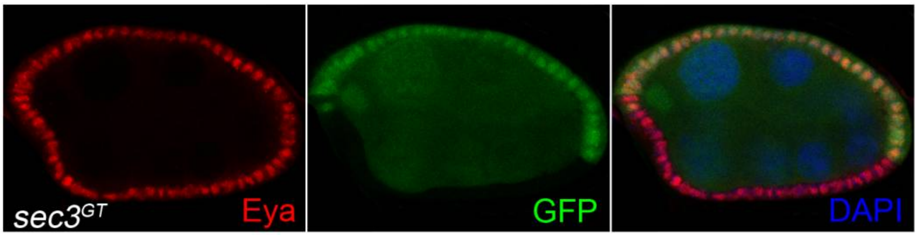

FIGURE S5. *sec3* mutant follicle cells show normal Eya expression in a stage 7 egg chamber.

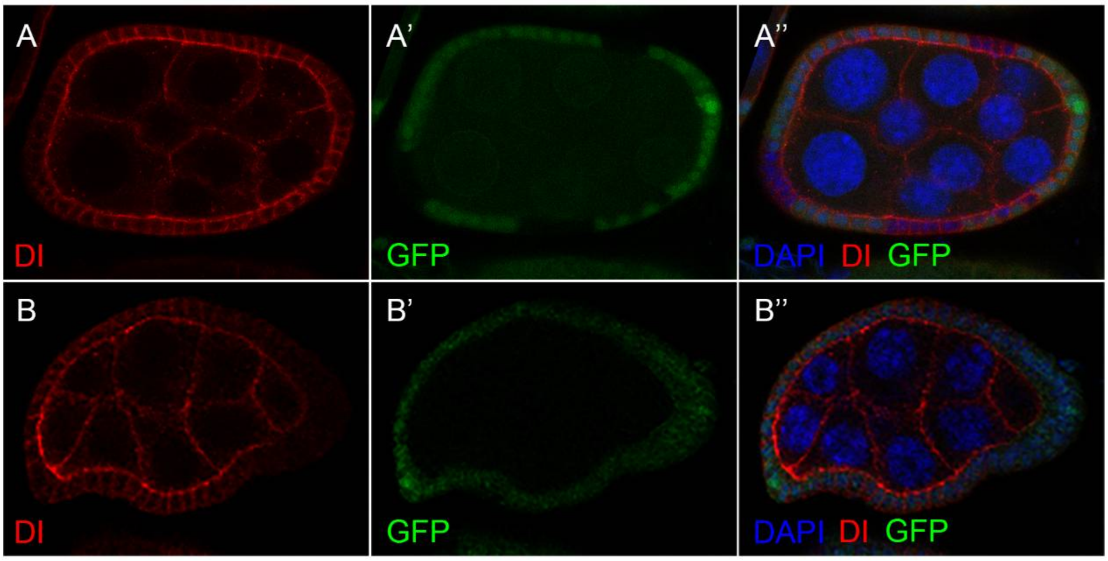

FIGURE S6. The distribution pattern and amount of Delta is normal in *sec3* mosaic egg chambers. (A-A'') Delta is not accumulated in *sec3*^{GT} follicle cell clone. (B-B'') In the differentiation of follicle epithelium during *Drosophila* oogenesis, Notch functions in the follicle cells while the ligand Delta activity is required in the germline cells (Lopez-Schier et al., 2001). Delta is not accumulated in *sec3*^{GT} germline cell clone.

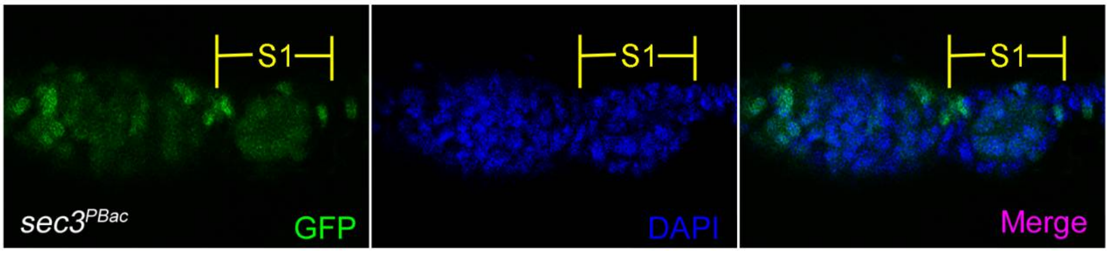

FIGURE S7. *sec3* mutant follicle cells can encapsulate germline cysts normally at stage 1.
